# Mr. Want, on the Treatment of Gout

**Published:** 1811-09

**Authors:** John Want

**Affiliations:** Surgeon to the Northern Dispensary


### *For the Medical and Physical Journal.*

### *Mr. Want, on the Treatment of Gout.*

Ὑπολήψιν δε εκ τελε το παθος ανιαραν εκλησαλο κ̧ μηδὲ ὅλως ὑπο τεχνης ιαλρικῆς ἰαθῆναι ποlε δυνηθῆναι. ἐγω δέ φημι ὡς εἰ διαγνωσθεῖεν καλως αἵ τε διαφοραι κ̧ τα ειδη αυλης ὅσα τὲ και οἷα τυγχανη, ευθεραπευlος ἀν ῥαδιως ὑπο των ιαlρων γενησεlαι.

"An opinion has been entertained that this disease is not curable by the medical art, but, I affirm, that if physicians make a proper distinction between the different species of the disease, it may *very easily be cured*."—ALEX. TRALLIAN, περι ποδαγρας, lib. xi.

"Est enim remedium hoc tantæ efficaciæ, ut hominem totum liberum a Podagra, et articulario morbo præservet, ita ut eo qui utitur, nihil ei opus sit de sanguine extrahendo aut remediis aliis, ut *longa experientia compertum habetur*." LUDOVICUS ENRIQUES, De Podraga.

NO subject of late has more engrossed the attention of the profession, and, perhaps, of the public at large, than the nature ture of gout, and the power of the French medicine in alleviating the paroxysms of that distressing malady. That a disease generally considered incurable should now be relieved, as it were, by a charm, could not fail to excite the wonder, and rouse the energies of the medical world. The effects of this medicine were no sooner acknowledged, than experiments were instituted in all quarters, as might naturally be expected, with the view of discovering its composition; but a period of nearly eighteen months had elapsed without any communication being made to the public upon the subject, until the appearance of Mr. Moore's Tract, in which he endeavours to prove its identity with a mixture of hellebore and opium. The reasonings in support of his opinion, as far as a cursory view of his paper has enabled me to judge of them, are conducted with much ingenuity; but whether he will succeed in establishing this point I shall not take upon me to hazard a conjecture. I must confess, indeed, that I attach less importance to this part of the inquiry than many may be disposed to give it. When first the medicine was administered in this country, when its effects were in a measure veiled in mystery, when more was attributed to it than subsequent experience proved to be warranted by fact, when it was supposed to possess properties totally unlike any other remedy in use, the inquiry was most natural. In the course of time, however, a long succession of facts has taught us so much of its operation as to supersede, in my opinion, the necessity of any new attempt to imitate it. Daily experience shews us, that a gouty paroxysm is curable by the production of certain sensible effects on the constitution. If any one medicine, therefore, or combination of medicines be discovered, which is capable of producing those effects, it is a fair inference that the gout may be cured with as much certainty in the one case as in the other, and the scientific physician will have no cause to regret his inability to compound a medicine, whose colour, taste, and smell, are similar to that which led him to the discovery. Under these circumstances it is only as a matter of curious speculation, that I should consider the farther prosecution of the inquiry as deserving attention.

Should Mr. Moore's opinion of this medicine, and the source from which it is derived, be supported by additional evidence, the profession will learn to set a higher value on the labours of antiquity than they have hitherto done. Their valuable repositories, in general, record the language of experience; and though much which they contain has been rendered useless by the efforts of succeeding generations, there yet remains a copious harvest for the industrious investigator.

From

From all that we can collect respecting the sensible operation of the gout medicine, it appears evidently to be a purgative of the drastic kind; and as most purgatives in an over dose evince an emetic quality in a greater or less degree, so upon this supposition it is easy to conceive that this medicine may, in many cases, produce nausea and vomiting.

Now the treatment of gout by strong purgatives is by no means a modern discovery. The limits of a paper of this kind will not allow me to trace back the history of the practice; it will be sufficient to observe, that it prevailed pretty generally and successfully previous to the time of Sydenham, when unfortunately for science and the afflicted sufferer, that enlightened physician interdicted the practice because it did not coincide with his ideas of morbific matter, and the necessity of its expulsion through the medium of the extremities. "It being," he observes, "an inviolable law of nature that the matter of the disease should be thrown out by the extremities; emetics and cathartics will have no other effect than that of bringing back the offending matter to the bowels."*

In another part of his treatise, we are told that the practice, however dangerous, was in high repute with certain empirics; he admits that during the purgative operation of the medicines administered the patient felt no pain, or at least very little; and that if the catharsis could be kept up for several days, the patient was certainly cured of that paroxysm.†

That this theory was framed in the face of personal experience, may be inferred from the latter part of the quotation, which amounts to an acknowledgment of the utility of the practice. That it was in opposition to the best practical authorities the world ever produced, will be evident to any one

---

* "Deinde, catharsin, quod attinet, sive *ἄνω* sive *κάτω* animadvertendum est, quod cum naturæ lex sit inviolabilis, atque ipsi hujusce morbi essentiæ intertexta innexaque, morbi fomitem semper in articulos rejici debere; nihil prorsus aliud præstabunt remedia sive emetica, sive cathartica proprie dicta, nisi ut materia peccans, quam natura in corporis extremitates protruserat, in sanguinis massam denuo revocetur; unde accidit ut quæ in articulos eliminari debuerat, in aliquod e visceribus forte irruat, atque ita æger, qui in nullo prius discrimine versabatur, jam de vita periclitetur."

SYDENHAM, Tractatus de Podagra.

† "At vero hæc ipsa methodus, utut perniciosa ac nocens, nihilominus empiricis quibusdam, qui catharticum quo utebantur medicamentum astute omnes celarunt, haud mediocrem estimationem conciliavit. Observandum est enim quod purgatione currente, æger vel non omnino, vel remisse admodum, dolet; & si catharsis ad plures dies produci queat, nullo superveniente paroxysmo recenti, æger confestim ab eo, quo jam tenetur, convalescet: verum enimvero pœnas in posterum pendet dirissimas ab *ἀλαξια*, in quam dicta humorum exagitatio naturam præcipitem egit."

who who will take the trouble to investigate the subject. Dr. Cheyne, says, " that in his time some eminent physicians had so little regard to the opinion of Sydenham in this matter, that in the fit of the gout itself *they never scrupled to drive it off*, both in themselves and others, by strong, quick, and active purges;" he further adds, " and most certain it is that this method will cure any fit of the gout, how obstinate soever, and that in a few days. We have also the authority of Kirkland for this practice, who asserts, that he has never yet seen under an inflammatory state, any method equal to purging for giving immediate relief; and this remark extends to the disease when it has attacked the stomach and bowels.

Scammony is a medicine which has been famed in all ages for its power of subduing the paroxysms of gout; and it is remarkable, that I have met with no author on the subject, whose prescriptions are not found to contain a large portion of this drug. The electuarium caryocostinum, the active material of which is scammony, was sold, in the time of Tissot, as a quack medicine in Geneva, under the title of Opiate for Rheumatism, and which he highly extolls with some exceptions as to particular constitutions. Bate recommends a tincture of scammony as a medicine of great service in the Gout, and the same tincture is, at this time, a popular remedy for that disease in France; and I am credibly informed that one gentleman for 13 years past has by means of it succeeded in removing the paroxysms whenever they occurred.

Salmon extols scammony and elaterium as medicines which " *admirably help* the gout," and Turner's arthritic powder is a composition of turbith, hermodactyls, scammony, senna, and elder seeds, and was considered in its day as almost a specific for this complaint.

Willis recommends the electuarium caryocostinum and hermodactyls, which, he says, will make such as are not able to go, presently to walk about. The same medicine was before prescribed by Alexander, with the same expressions, as to its virtues, so as to leave little doubt of its having been taken from that author*. He quotes Rodericus a Fonseca in recommendation of the roots of black hellebore, and amongst other things an apple, with ʒss of its fibres stuck in it, which is to be roasted under the embers and eaten.

It would be easy to multiply authorities if it were necessary,

* Alexander's prescription is composed of hermodactyls, ginger, cummin seed, pepper, aniseed, and scammony, which makes those who take it walk immediately.

" τουτο ευθεως βαδιζειν αυτους ποιει."

Alex. Trall. in loco.

but but as they are within the reach of every inquirer, I shall merely add the following extract from Hoffman, which, from the information it contains, appears of too much importance to be omitted.

"Hisce deductis operæ pretium esse estimo paucis judicium nostrum interponere de methodo hactenus usitata, sanandi dolores podagricos. Plures et ex iis exercitatissimi practici vomitorium usum non satis dilaudare queunt in affectu hoc, tam preservando quam sanando. Confert, ut podagrici omni verno et autumnali tempore circa æquinoctia preservationis causa assumunt vomitorium. In principio quoque podagræ, proficuum esse ipsi Galenici jamdudum testati sunt. Dolæus Encyclop. Med. p. 635. Hildanus, Cent. 6. Obs. 34. Mercatus de Morbis internis, L. 4. c. 18. Riverius praxi, L. 6. c. 1. Sylvius, pr. p. 153. Emeticorum usum præsentissimum judicant. Alii magni faciunt preservationis causa laxantia ante ipsum paroxysmum, vel singulis mensibus.

"Doctissimus Solenander in Cons. p. 78, scribit se in paroxysmi principio solere exhibere, magno cum successu, aliquod purgans; primum id edoctum ab Alex. Tralliano, l. xi, licet a multis hoc tempore reformidatus. Singulare esse scribit eruditissimus Pechlinus, Obs. 26, quod arthritici leviorem plerumque experiantur insultum, detracta ex primis viis materia. Novi, ait, Empiricum qui medicamento chymico, seu Chrystalli Lunæ essent seu Martis aliquod vitriolum, etiam in inveterato jam morbo postquam biduo ante paregoricum dedisset, alvum magno cum fructu provocabat. Ex adverso Sydenham illorum usum penitus rejicit. Nostra sententia hæc est qui plenius vescuntur, et largiori victui assueti sunt, et mox ante paroxysmum primum invitante appetitu plus justo indulserunt gulæ appropriata cathartica vel emetica summo cum fructu atque emolumento assumere possunt. Depletis sic primis viis a copiosa saburra humorum et acidi superantis, leviores evadunt paroxysmi, nec tam molesta symptomata anxietates dolores patiuntur ægri, &c. In eandem sententiam nobis cum accedit Lister, Tract de Arthridite, qui ita loquitur, &c."*

---

* Dissertatio de genuino et simplicissimo podagræ remedio. Hoff. oper. vol. vi.—The sense of the author may be thus rendered:—"I shall say a few words on the method hitherto employed for the cure of gouty pains. Many of the most experienced practitioners speak in the highest terms of emetics in this complaint, both for prevention and cure. Gouty patients derive great benefit from emetics taken as prophylactic, at the vernal and autumnal equinoxes. In the commencement also of gout, the beneficial effects of these remedies have been long ago testified by the followers of Galen. Dolæus, Heldanus, Mercatus, Riverius,

In another treatise, de dolore podagrico et arthritico, he says, "Memini me olim statim subinitio doloris podagrici et arthritici, insigni sæpe cum fructu, usum fuisse sequentis mixturæ. Aq. flor. acaciæ ℥ij. Aq. cinn. sine vino ℥ss. P. cornachini ʒj. Syr. rosar. laxativ. vel rhabarb cum cichoreo ℥ss. M. pro haustu. A dram of the pulv. cornachini contains, scammony, diaphoretic antimony and cream of tartar, of each equal parts, so that twenty grains of scammony was given for a dose.*

The experience of the present day is equally strong in favour of the practice.

Very early during the agitation of this question the attention of the profession was generally directed to the Elaterium as a medicine most likely, from its known effects, to be the basis of the *Eau Medicinale*. I determined, therefore, to put it to the test of experiment on the first patients I met with. Three cases soon presented themselves, in which the curative powers of this remedy were as speedy and as satisfactory as any thing that could possibly have been expected from the gout medicine itself.

It is deserving of remark, that independently of its purgative, emetic, and diuretic effects, Elaterium possesses another property in common with the gout medicine, that of producing a tingling sensation in the extremities of the fingers.

---

rius, and Sylvius, consider emetics as a most effectual remedy. Others think highly of laxatives as a means of prevention before the paroxysm, or taken monthly.

"The learned Solenander writes, that he was in the habit of administering a purgative with great success, in the commencement of a paroxysm, that he first learned this practice from Alexander of Tralles, though by many it was much dreaded.

"Pechlin says, 'it is a singular fact that arthritics have a milder fit if the primæ viæ have been first cleared. I know,' says he, 'an empiric who, by a chemical medicine, whether it was the chrystals of silver, or a vitriolate of steel, is uncertain, procured evacuation from the bowels with great success, and this even in an inveterate disease, he having given a paregoric two days before.' On the contrary Sydenham entirely rejects the use of purgatives. I am of opinion, that in those patients who are accustomed to eat much, and who just before the paroxysm have been too indulgent with their appetite, proper emetics or cathartics may be taken with the greatest advantage.

"The primæ viæ being thus cleared of the suburra of the humors and superabundant acid, the paroxysms become milder, nor do the patients feel so much pain or uneasiness. Lister, in his Treatise on Gout, is of the same opinion with me on this subject, and has these words, &c."

* "I remember having used the following mixture with great success in the commencement of gouty pains, &c." De dolore arthritico et podagrico. Hoff. opera, vol. 2.

This effect, which was noticed in the year 1695 by Dr. Martin Lister, was first pointed out to me by Dr. Buchan of Percy-street, and was very strongly marked in the persons to whom I administered it. Whether this property is only exerted in gouty patients must be determined by future observation; Dr. L. however, spoke of its effects generally in his Treatise on Dropsy*.

The first case which was submitted to its influence was that of a labouring man, John Tomkins, who had long been a sufferer from that form of the disease called by many rheumatic gout†, and was never entirely free from its attacks; he had been under the care of the most eminent Hospital physicians in town without deriving any benefit from their treatment. I recollect having attended him upon a former occasion under a severe illness from this complaint, when his life was despaired of for many days, so much was he reduced by its violence and duration; at this time, the stomach and bowels were much affected by metastasis, and he recovered under the use of large doses of volatile tincture of guaiacum‡.

He had now inflammatory swellings of several joints, particularly of his hands, wrists, and feet; the knuckles were permanently enlarged, and the fingers in a state of contraction, from former attacks. The pain often subsided in one part for another; sometimes it went from the joints to the bowels, and his friends became seriously alarmed for his safety. I gave him half a grain of elaterium every two hours, and on the following morning I found him free from all his symptoms, but great debility, and complaining of the severe operation of his medicine. The disease returned the following day and was again relieved in the same manner.

Within these few days, a period of nearly eight months having elapsed since his last illness, he has had another attack of the gout; but here the seat of the disease was not as in the other case in the extremities, but confined to the

---

* Lister Exercitationes de Morbis Chronicis. Tract. de Hydrop. p. 50.

† Some practitioners call the acute rheumatism, rheumatic gout; the latter disease, which is here alluded to, is characterized by inflammatory swellings in the larger joints of the body, *with little or no fever*, whereas acute rheumatism is always attended with great heat of skin, and the other symptoms of fever.

‡ A more extended experience, since this time, has abundantly confirmed me in the opinion I had formed of the efficacy of this remedy when administered in large doses. I think I may assert with confidence, that there are few cases of Gout which may not be subdued by means of it. The efficient dose is ʒss or ʒvj taken in half a pint of water gruel night and morning.

bowels. He had been ill nearly a fortnight, complaining of tightness across the bowels, and what he denominated a heavy pain; he had nausea, impaired appetite, and flatulency; his bowels were costive, and he was generally indisposed. These were considered to be symptoms of gout, affecting some of the abdominal viscera. Three grains of elaterium were ordered, with directions for him to take one half immediately, and the remainder at night. Having taken one portion of this medicine, in the evening he was attacked with the most violent pain in his bowels; the remaining portion was given him, and a purgative glyster with two drams of aloes and the same quantity of jalap ordered, which however was not administered. On the following morning I found he had been in excruciating pain all night; in the evening I saw him, the tongue was of a reddish brown colour and parched, his breath offensive in a remarkable degree, of which no words can convey an adequate idea, but which is very commonly met with in this stage of the complaint. With these symptoms of evident danger I did not think it right to rely solely on the elaterium: three grains more were given, and ℥xvj of blood ordered to be taken away; he was easy for the space of three hours after the bleeding, when the pain returned; on the following morning he was in great pain, the medicines had not operated. The glyster was repeated twice this day without effect; in the evening he was still in acute pain; and was again bled; ℈j of calomel was given, with directions to have the glyster repeated, which however was omitted; he was much relieved by the bleeding, but on the following morning the pain returned; he had one stool in the night; half an ounce of volatile tincture of guaiacum with fifty drops of laudanum were now given, which produced ease for the whole of the day. Towards evening the pain returning, the medicines were repeated with equal benefit. On the following morning he was free from pain, and his bowels were open; he was directed to continue his medicines a day or two longer, and is now quite recovered.

In the other cases the disease was confined to the back of the hand and wrists, it had here existed many days; each had three grains of Elaterium administered, which produced a strong purgative effect, but the symptoms were removed with the exception, in one case, of a trifling degree of pain still remaining, though it was not of sufficient importance to require a repetition of the medicine. In one of these patients it was remarkable that the pain ceased before the evacuation from the bowels took place; it is not improbable that the salutary operation of the medicine was occasioned by some change in the secretions of this viscus effected by it. The same result is sometimes observed from the Eau Medicinale.

 These

These cases have been related to shew the advantage of attacking this complaint by purgatives. The precursory symptoms of a paroxysm of gout; the inappetency, flatulence, sour eructations, and constipated bowels, indicate a disordered state of the intestinal canal. If the village dame be consulted by a patient in this case, he will be told that his bowels are in a bad condition, that he must take a dose of physic; and, probably, the opinion of the medical practitioner will not be very different, if he has not the good sense to know that gout is coming on. But if his mind is warped by prejudice this knowledge is sufficient for the prohibition, and the unfortunate sufferer is consigned to patience and his flannels.

The *sicca alvus*, or torpor of the intestinal canal, is a very common attendant on this disease. The celebrated Father of Physic, who lived 360 years before Christ, thought that gout was curable by no human means in old men, with *constipated bowels* and nodosity of the joints. But the disease even in them was not proof against a providential attack of dysentery, which cured it readily. He found other profuse evacuations from the bowels (εκτηξις, which literally signifies a melting down) also very useful; and he tells us, that a young man whose bowels were readily acted upon, who lived regularly and was fond of labour, had only to find a physician with common understanding and he might easily be cured.*

The power of diarrhœa in carrying off a fit of the gout when seated in the extremities is so notorious, that it could hardly escape so accurate an observer as Sydenham; but blinded as he was by a prepossession for his favourite opinion, he could not be expected to derive that practical advantage from it which the fact was capable of imparting. We cannot then be surprised to find him giving solemn directions for its suppression, as the only means of recalling the wanderer to his proper seat.

"There is but one remedy," he says, "that I know of, viz. to produce perspiration, by the medicines usually given for this purpose, which if effected for two or three days morning and evening, two or three hours at a time, the diarrhœa is commonly stopt, and the gout comes *thundering* back upon the extremities.*" It should be remarked, however, that when he has reason to believe the diarrhœa is critical, he does not attempt to interfere with it.

It is impossible for the imagination to contemplate the strength of the *thundering* figure, which he uses to express the return of gout to the limbs, without picturing to itself the triumphant extacy of the man on the seeming victory he had obtained; whilst his patients, writhing under the most insufferable anguish human nature is capable of bearing, are consoled with the cheering prospect of longevity, which he invariably calculates upon, *cæteris paribus*, in a certain proportion to the violence of the pain.†

How different is the conduct of Musgrave on this point, who after telling us that during a paroxysm of gout a diarrhœa often takes place, which carries off the pain and tumefaction‡, adds, that "the diarrhœa which *anticipates* the fit is frequently found to be salutary, health and vigor returning after it.§ The event of it, however, he confesses is uncertain. If it stops in time and is not *excessive* it proves serviceable, by carrying off the gouty matter by a safe way, though not the most common; and it has this advantage attending it, that the paroxysm *does not return for a long time after*.|| He displays the folly and danger of an attempt to stop it; "for an officious diligence," he says, "disturbs nature, and interrupts her in the work she has begun, when it is better to leave her to herself, and permit that to be dis-

---

* "Unicum, quod scio, remedium est, ut sudor provocetur methodo et medicamentis huic usui destinatis: quod si fiat ad biduum triduumve mane & vesperi, per duas tresve horas continuas, sistetur ut plurimum diarrhœa, & morbi fomes magna vi in artus *detonabit*." SYDENHAM.

† "Morbo jam discusso, ægri tum εὐεξία, tum appetitus, redeunt, pro rata doloris quo sæviebat paroxysmus nuper elapsus, & in eadem proportione servata, vel acceleratur vel differtur sequens paroxysmus. Nam si hic ultimus ægrum *pessime multaverit*, sequens paroxysmus non, nisi anno ad idem punctum revertente, denuo accedet."—

SYDENHAM.

‡ "Accidit diarrhœa; eamque statim excipit doloris et tumoris diminutio." De Arthritida Anomala, page 137.

§ "Atque istiusmodi fluor paroxysmos interdum Arthriticos antevertens, salutaris esse reperitur. *Expulso etenim inimico, clauduntur januæ; pax, et quies restituitur; viget œconomia.*" Page 138.

|| Hujusmodi diarrhœa incerti admodum eventus est; si enim tempori cohibeatur, neque modum excedat, utilis invenitur; et quidem arthritidis materiam etiamsi via non usitatissima, tamen tuta emittere. Cui isthuc consequens, quod paroxysmus *non nisi diu post* recurrit arthriticus. P. 139.

charged, charged, which if retained would do mischief; but if it becomes excessive it should certainly be restrained.*

That the gout is symptomatic of the affection of the bowels, I have little doubt. Stahl endeavours to account for the alternation of this disease with hypocondriasis, which may evidently be traced to this source.† If we admit that erysipelas is often connected with sordes in the intestinal canal, we shall have little difficulty in conceiving that another inflammation, in some respects similar, may arise from the same cause.

The affinity that exists between these two diseases may be inferred from the fact of their frequently alternating with each other. After a paroxysm of gout has been fairly fixed upon a joint, in all regularity and form, and has continued there for several days, it is sometimes suddenly transferred to the face and neck, or other parts, where it appears in the shape of an erysipelas, and *vice versa*.

Hoffman relates the case of a patient who had an extensive erysipelas of the right leg, which soon seized the metatarsus and toe of the left side, with considerable tumefaction and pain. The surgeon, who was in attendance, applied spirit of wine and camphor to the foot, upon which it attacked the other: by the application of powdered camphor, the disease was again immediately relieved, but the patient was seized with symptoms of gout in the vicinity of the heart, with so great a constriction upon the diaphragm that he could hardly breathe.‡

Musgrave also speaks of the transition from erysipelas to gout in the joints§; but he is not warranted, I think, in assuming from this fact the existence of an arthritic erysipelas. The *morbi fomes*, the daily accumulations of indigested sordes, is the exciting cause of both these and of many other diseases. Gout in the joints, erysipelas in the skin, asthma, phthisis, peripneumony in the lungs, colic in the bowels,

---

* Nihil inepta sedulitate *stultius* dicam an *periculosius?* quæ, Pharmacis perperam ingestis, naturæ opus sæpe interturbet, interdum abrumpat. Suo potius arbitrio ea, patiaris, agat; quodque intus læsurum erat suo utiliter ingenio effundat, si vero diarrhœa *nimium excurrat*, viresque excedat, astringentibus sistenda est. Page 139.

† Stahl. Theoria Medica, page 1372.

‡ Observations Interessantes sur la Goute & Rheumatism, traduites de Fred. Hoffman.—Obs. 2. 241.

§ " *Faciei* porro *erysipelas* notavi quod detractione sanguinis, factaque inde materiæ in artus Διαδοχῆ quuum in *dolorem* mutaretur isthuc *arthriticum*, naturam plane confessum est *arthriticum*, &c."—Musgrave de Arthritide anomala, p. 459.

commonly commonly called gout in the stomach: these, and a variety of other symptoms from the same cause, may be removed by the evacuation of the offending matter, or by retunding and neutralizing its acrimony. His book is a most invaluable production, inasmuch as it shews what an endless variety of nervous and chronic diseases may be traced to this source.

If this opinion be established, the superiority of purging over bleeding will be evident; the latter may relieve, as in all cases of inflammation it does, but it is only by removing the cause that we can expect a removal of the disease.

With these facts before me, I cannot help recurring to the exploded doctrine of the humoral pathology, as affording the only plausible solution of the phenomena of this malady. Is it, I would ask, inconsistent with the known laws of the animal œconomy, to suppose that an acrid matter may be carried by absorption from the intestines into the circulation, and that its existence in the blood is incompatible with the healthy functions of the body? If I might be allowed to step beyond the limits of fair induction into the field of hypothesis, I would imagine the joints, from some peculiarity unknown to us, to be the parts chosen by nature for the deposition of this matter, or I see no difficulty in allowing its transpiration to be going on through every pore, whilst the joints or parts attacked, from this unknown peculiarity, are more susceptible of diseased impression from its acrimonious nature than others.

Pain is not an inseparable concomitant of this disease; its intensity, or indeed its very existence, is probably determined by the texture or sensibility of the parts attacked. Thus in the commencement of a regular paroxysm which is seated in the ligaments of the joints, acute pain is felt, but when at the close of each "*paroxysmulus*" the pain is commuted for swelling, erythematous redness, and itching of the surrounding integuments, it is not unfair to conclude that the matter has been translated from the ligaments to the skin. When the disease by metastasis or otherwise affects the brain, separate from its meninges, no pain is felt, but giddiness, stupor, or apoplexy are the consequence.

Cullen's fourth objection to the theory of morbific matter is, that its operation, if it exists, should be similar in the several parts which it attacks; whereas it seems to be very different, being *stimulant*, and exciting inflammation in *the joints*, but *sedative*, and destroying tone in *the stomach*.*

Lest any doubt should arise of the truth of this position, from the natural insensibility of parts of a ligamentous struc-

---

* First Lines, Sect. 529.

ture, ture, I must be allowed in digression to observe, that however insensible in their healthy state, when they have taken on inflammatory action the pain is most *acute*. This arises from their dense, firm, and unyielding nature.

The acknowledged efficacy of blisters* in the cure of gout, in many cases, when applied to the inflamed joint, strongly favour the theory of morbific matter. No person in the right possession of his senses would think of curing inflammation by means of blistering, unless with the hope of exciting a discharge of some irritating material producing the disease.

The local application of euphorbium,† elaterium,‡ and medicines of this kind, the beneficial effects of which are attested, the former by Trallian, the latter by Dioscorides and Pliny, can hardly be accounted for upon any other supposition.

Another reason in support of this theory, to say nothing of the relief demonstrably given by purgatives, is the immediate benefit arising from the use of those medicines which have the power of neutralizing acid secreted in the stomach and bowels. Half an ounce, or a larger dose of volatile tincture of guaiacum, given night and morning, is a remedy which from much experience I can recommend as a specific in many cases of this disease.

If the medical reader requires more evidence than his own experience will afford him of the existence of this acid, I may refer him, amongst others, to a case of gout related in the first volume of the Medical Observations and Enquiries, where a severe paroxysm was critically carried off by the vo-

---

"I have seen another patient who, by means of a medicine composed of cantharides, has been cured, for when the vesication occasioned by it was ruptured, there came forth a great quantity of humour, which he said relieved him greatly." Alex. Trallian.

"It is a noble medicine, and frequently tried with success in this species of the complaint, for it excoriates and removes the cuticle, it draws forth that which is deep seated, and relieves the pain."

Alex. Trallian.

‡ "Radix elaterii ex aceto cocta podagricis illimitur succoque dentium dolori medetur."—Plinii Hist. Nat. de Elaterio.—Dioscorides is literally copied in this quotation.

miting mitting of a considerable quantity of fluid, which from its acrimony was compared to strong mineral acid; added to this, the close analogy which subsists between some forms of dyspepsia in which the presence of acid is indisputably certain, and those symptoms which precede an attack of gout, leave little doubt of its existence also in the latter case.

In a systematic treatise much more might be adduced in support of this opinion; but as I fear I have already trespassed too long upon the patience of the reader, I must remain satisfied with having merely laid the foundation for future inquiry.*

If we admit this fact, the dangerous consequences of an indiscriminate attempt to cure gout by the application of cold water will be obvious. That in many cases the powers of nature are proof against the destructive tendency of this practice cannot be denied, but that fatal instances have occurred in consequence of its employment is equally certain. Upon this point Trallian judiciously observes, "that no astringent or repellent medicines should ever be had recourse to, until the bowels have first been freed of the sordes contained in them.†

---

* The objections to this doctrine first started by Stahl, and afterwards urged by the celebrated Cullen, are of little weight in the scale when opposed to the host of evidence which may be adduced in its favour. Cullen says, we have no direct evidence of the existence of morbific matter in the blood, and that previous to an attack of gout there appears no marks of any morbid state of the fluids. Granted: but we have strong proof of its existence in the intestines; and we shall have more difficulty in supposing the possibility of its continuance in the bowels without being absorbed into the circulation, than in accounting for the mode of its getting there. The arguments against the existence of this matter, deduced from the various and contradictory accounts of its nature, are too futile to deserve a moment's attention. It can never be deemed an objection to the truth of an opinion, that its partizans have taken wrong measures in its defence. Hence the attack upon the doctrine contained in his 6th objection, founded on the mistaken inferences of its existence, from the hereditary nature and contagious property of the disease, is equally absurd with that last considered. The only objection which remains unanswered is, that the theory is inadequate to explain its frequent metastases from one part to the other. Is Cullen's theory unimpeachable on this score? No. Admitting then that we are not sufficiently informed respecting it to be enabled to explain *all* things *at present*, let us take that which we have as an earnest of that which is to come; and having nothing more plausible to oppose to it, wait with patience until more facts, or some newly discovered analogy, afford the complete and desired explanation.

ALEX. TRALL.

That gout is curable by medicine and proper attention to diet and mode of living, without danger of inducing other diseases, I have long been convinced by great attention to the subject; and so fully persuaded am I of this fact, that I should feel no hesitation in affirming that it is so in every case, if the experience of one individual could afford a sufficient warrant for such an assertion.

The administration of purgatives forms a prominent, though not the only feature in the medicinal part of its treatment. Their employment we have shewn to be warranted by facts; they have the voice of reason on their side, for when "the enemy is driven from the citadel, and the gates shut, peace and tranquility *must* be restored." They have the sanction of the most unquestionable authorities of antiquity for their utility; but that they are capable of removing the disease in every case, is more than I dare venture to affirm. Many cases may, no doubt, occur, in which they may not only be inefficient, but dangerous. The discrimination of the practitioner must here be exercised; Elaterium, though in a proper dose a *mild* medicine, will destroy life as well as the Eau Medicinale, and it is only by minute attention to the circumstances of the case, that its employment can be regulated.

As to the identity of any preparation of Elaterium with Husson's remedy, it is, as I hinted above, of too little importance to contend for. Yet, I may observe, that the marked coincidence in effect between these medicines, speaks as strongly in its favour as medical evidence can speak. What pharmaceutical process it should undergo to render it similar in external appearance to that medicine, I shall leave to the consideration of those who are more curious in researches of this nature than I am.

This is a point at which no man of science need be ambitious of arriving. The research is endless. The time which has already been employed in the inquiry, and the varied speculations that have been entered into respecting it, with so little success, will almost justify this conclusion. It appears to me to be a prostitution of talents, implanted in us by the great Author of our being for wiser purposes; for we are seeking for that, which when discovered will add comparatively nothing to the stock of our information.

If I have shielded myself under the authority of men whose names are venerable in the annals of Medicine, I trust I have not suffered myself, by a blind adherence to them, to be drawn into the track where experience does not also lead. The remarks here made might have received support from cases under my own observation, but it is easy to make a tale suited to any theory, and the narratives of some modern writers are so so much in opposition to the daily experience of all practitioners but themselves, that I was unwilling to risk an imputation so disgraceful to a man of science.

It is with reluctance that I have been induced to launch out into theory; contrary to my original intention, I have insensibly been drawn into it. If my opinions are opposed to facts, I shall be most happy to cancel them and to acknowledge my error; I have no object in this investigation but truth, and if I have not yet found her, she shall be a welcome guest whencesoever she may come.

JOHN WANT,
Surgeon to the Northern Dispensary.

*July* 23, 1811.

